# Association Between Refrigerator Openings and Protein Intake After Hospitalization for Heart Failure Decompensation: Protocol for a Prospective Cohort Pilot Study

**DOI:** 10.2196/66299

**Published:** 2025-08-18

**Authors:** Karin Haas, Franziska Scheidegger-Balmer, André Meichtry, Benjamin Vögeli, Nisha Arenja, Philipp Buluschek, Hugo Saner

**Affiliations:** 1 Department of Health Professions Division of Nutrition and Dietetics Bern University of Applied Sciences Bern Switzerland; 2 Department of Health Profession Bern University of Applied Sciences Bern Switzerland; 3 Department of Cardiology University Hospital of Basel Basel Switzerland; 4 Kantonsspital Olten Solothurner Spitäler AG Department of Cardiology Olten Switzerland; 5 DomoHealth SA Lausanne Switzerland; 6 ARTORG Center for Biomedical Engineering Research University of Bern Bern Switzerland; 7 Institute for Social and Preventive Medicine University of Bern Bern Switzerland

**Keywords:** ambient sensor system, digital health, older people, muscle mass, muscle strength, nutrition assessment, protein intake

## Abstract

**Background:**

Sarcopenia, characterized by loss of muscle mass, reduced strength, and increasing frailty, is common in older adults and is often the result of underlying diseases such as advanced stages of heart failure (HF). Protein intake is crucial for maintaining muscle mass and strength. However, older adults typically consume less protein compared to younger individuals. It has recently been shown that the time of the first refrigerator opening in the morning, indicated by a door sensor, may be correlated with frailty in older individuals living alone.

**Objective:**

The aim of this study is to determine whether the time of the first refrigerator opening in the morning can serve as a potential indicator of protein intake in older patients (age ≥70 years) after hospitalization for HF decompensation over a period of 6 months.

**Methods:**

This is a substudy of a prospective interventional cohort study that aims to identify changes in digital biomarkers derived from an ambient sensor system, which may support the early detection of HF decompensation. In this substudy, the frequency and timing of refrigerator door openings in participants’ homes will be measured. To assess associations between protein intake and refrigerator openings, dietary intake will be evaluated using 24-hour dietary recalls at 3 different timepoints: at 1, 3, and 6 months after hospital discharge. All 24-hour dietary recalls will be conducted by trained dietitians in face-to-face interviews on weekdays. The primary outcome of this study will be the correlation between protein intake and the time (in minutes after midnight) of the first refrigerator door opening at each of the 3 assessment points.

**Results:**

The study is currently in the data collection phase. Recruitment began in February 2024. Data analysis will begin after all data have been collected. As of manuscript submission, 12 patients have been recruited. Results are expected to be published by the end of 2025.

**Conclusions:**

Considering that protein-rich foods are typically stored in the refrigerator, the relationship between frailty and refrigerator usage may be a relevant indicator for nutritional assessment, particularly regarding protein intake. In addition, sarcopenia and frailty may also be linked to protein distribution across meals. This study will explore whether monitoring refrigerator openings can serve as a simple method for identifying increased risk of frailty and sarcopenia. Early detection through such monitoring could facilitate timely interventions and potentially reduce the risk of these conditions.

**Trial Registration:**

ClinicalTrials.gov NCT06126848; https://clinicaltrials.gov/study/NCT06126848

**International Registered Report Identifier (IRRID):**

DERR1-10.2196/66299

## Introduction

The population of older people is increasing significantly around the world, including in Switzerland [1]. With higher life expectancy and increasing age, the risk of diseases characterized by a decline in muscle mass is also rising [2,3]. Loss of muscle mass and strength is associated with an increased risk of mobility impairment, fractures, prolonged hospitalization, hospital readmission, reduced quality of life, morbidity, and mortality [2,4-6]. These conditions arise due to a combination of reduced food intake, increased nutrient requirements, metabolic changes, sepsis, trauma, aging, and physical inactivity [4]. The interplay between malnutrition, sarcopenia, and frailty in older people has already been demonstrated in many studies [7-10]. A systematic review and meta-analysis has shown that about half of hospitalized older adults suffer from 2 or even 3 of these diseases or precursors [3].

Furthermore, sarcopenia and frailty are often the result of other underlying diseases such as chronic heart failure (HF). The prevalence of sarcopenia has been found to be 55% (95% CI 43-66) in hospitalized patients with HF and 26% (95% CI 16-37) in nonhospitalized patients [11]. Additionally, 79% of patients with HF are frail. This, in turn, may be associated with reduced quality of life and poor prognosis [12]. Overall, diet quality is associated with the risk of frailty, and poor intake is adversely related to muscle mass and strength [13,14]. Nutritional status is, therefore, also a crucial risk factor for the development and prognosis of HF.

Due to the high prevalence of sarcopenia and frailty in patients with HF, more attention should be paid to protein intake as an important influencing nutritional factor [14-16]. To achieve this, the protein intake of patients with HF in the home environment must first be assessed. Ideally, simple, valid instruments should be used for this purpose. Common screening tools such as the Mini Nutritional Assessment (MNA) are insufficient to recognize people with low energy and protein intakes [17]. Currently, a number of screening and assessment tools are available [18]; however, the assessments with these tools are very time-consuming for long-term monitoring at home and therefore not very feasible for application in HF patients’ everyday lives.

Digitalization can offer new possibilities that allow patients with HF living at home to be monitored easily and regularly. It has been shown that a sensor system using interaction-free, contactless, and inexpensive sensors is suitable for long-term monitoring of physical activity, sleep, and door openings, including the refrigerator door in the homes of older people living alone [19]. Considering that protein-rich foods (such as meat, meat products, fish, milk, and dairy products) are typically stored in the refrigerator, the relationship between frailty and use of the refrigerator door could be an important indicator for nutritional status in terms of protein intake. Furthermore, sarcopenia and resulting frailty may also be linked to protein distribution across meals. To date, very few studies have investigated this. A study among older women in the Nurses’ Health Study indicates that consuming at least 30 g of protein over 2 or more meals could be more effective for maintaining muscle mass and physical performance compared with eating a single high-protein meal [20]. In addition, 2 recent studies in older populations indicate that skipping breakfast increases the risk of frailty [21,22]. A Japanese survey (n=2468) showed a correlation between breakfast habits and frailty in older people aged 75 and over. Breakfast skippers generally had lower food intake and poorer nutrient density than daily breakfast consumers. The multivariable-adjusted odds ratio (95% CI) of breakfast skippers for frailty was 1.62 (1.04-2.52; *P*=.032) in one model (adjusted for age, sex, body mass index, and living alone) [22].

The use of refrigerator openings (eg, the first door opening of the day or frequency) as indicators for increased risk of sarcopenia and frailty would provide an easy monitoring opportunity. In this context, the study by Schütz et al has shown that the time of the first refrigerator opening in the morning indicated by a door sensor at the refrigerator may correlate with frailty in older single living persons [19]. Notably, approximately one-third of older people in Switzerland live alone [23], emphasizing the relevance of such monitoring approaches. Prior studies have also shown that living alone increases the odds of falling among community-dwelling older adults [24-26]. With such simple assessment tools, interventions could be initiated at an early stage to reduce the risk of these diseases. This, in turn, would have a positive impact on the quality of life of those affected and could also help to reduce health care costs.

As a part of prospective interventional cohort study (for details refer to study protocol of Vögeli et al [27]), a substudy will be conducted with the aim of evaluating the sensitivity and specificity of the time of the first refrigerator opening in the morning as a potential indicator for protein intake in older patients after hospitalization for HF decompensation over a period of 6 months.

## Methods

### Study Design and Participants

In this prospective cohort pilot study, 24 consecutive patients living alone at their homes who were hospitalized for HF decompensation at 2 secondary care hospitals (Solothurner Spitäler AG) in Olten and Solothurn, Switzerland, will be included.

The inclusion criteria are current hospitalization for HF decompensation, age≥70 years, left ventricular ejection fraction <50% and treatment with diuretics, New York Heart Association class II or III, living alone, willingness to participate with informed consent, and agreement to attend follow-up visits at the hospital at 3 and 6 months. Exclusion criteria include major depression (9-item Patient Health Questionnaire [PHQ-9] score >9) or being on hemodialysis. In addition, patients with a left ventricular assist device, or those who had undergone coronary revascularization or cardiac resynchronization therapy implantation within 28 days before the index event of HF decompensation, or have been scheduled for such interventions, are excluded from the study.

### Assessment

#### Baseline Assessment

The baseline parameters that will be evaluated at hospital entry have been previously described [27].

#### Dietary Assessment

For dietary assessment, food intake will be measured using the 24-hour dietary recall. The 24-hour dietary recall is an open-ended, retrospective dietary assessment method. It aims to record as much detailed information as possible about all foods and drinks consumed by the respondent in the previous 24 hours or the day before [28]. In this study, 24-hour dietary recalls are conducted at 3 different timepoints, at 1 month (optional), 3, and 6 months after hospital discharge. The first 24-hour dietary recall at 1 month is optional, as—unlike the assessments at 3 and 6 months—it cannot be coordinated with a routine follow-up hospital visit. Conducting this assessment would require an additional clinic or home visit, which was deemed burdensome for participants. To reduce participant burden and support adherence, this timepoint was made optional. All 24-hour dietary recalls will be conducted on weekdays by trained dietitians in face-to-face interviews. As the main drawback of this method is the forgetfulness of the participants, the 24-hour dietary recalls will be conducted by using the Automated Multiple-Pass Method (AMPM), as described elsewhere [29]. The AMPM uses multiple memory cues with standardized wording to elicit recall of all possible foods. Before the start of the 24-hour dietary recall, it will be recorded whether the day surveyed reflected a usual day in terms of diet or whether it was an unusual day. A usual day is defined as a day on which the participant ate according to their usual habits and routines, reflecting habitual dietary intake. This terminology was chosen to emphasize the importance of capturing habitual intake patterns. Conversely, an unusual day is determined based on the participant’s own judgment and refers to any day they perceive as not representative of their usual intake. Examples will be provided to participants, for example, holidays, celebrations, excursions, acute illness, or other events that may have significantly altered their eating behavior. The amount of consumed food will be estimated using the photo book of the National Nutrition Survey in Switzerland (menuCH) [30] (Multimedia Appendix 1). The conversion of the estimated amounts into weights will follow the menuCH photo book manuals and commercially available packaging sizes in Switzerland. All conversions will be performed by experienced dietitians. Protein intake in grams (g) and energy intake in kilocalories (kcal) will be calculated using nut.s nutritional software (version 1.33.10 - 2022.08.26; dato Denkwerkzeuge) based on the 24-hour dietary recalls. The nutritional database used in nut.s is adapted for the evaluation of Swiss food products.

#### Anthropometric Measurements

Anthropometric measurements include body height, body weight, and BMI. Weight is measured using a clinically validated and calibrated scale. Participants must remove heavy clothing (eg, jackets and jumpers) and shoes. Weight is recorded to the nearest 0.1 kg. Height is measured using a calibrated stadiometer. The measurement is taken in an upright position with the feet on a hard surface. The heels, buttocks, shoulders, and the back of the head touch the wall, and the arms are placed at the sides. Height is recorded to the nearest 0.1 cm. BMI is calculated by dividing body weight in kilograms by height in meters squared. Measurements will be taken at 1 month (optional), 3, and 6 months after hospital discharge, each time following the 24-hour dietary recall.

#### Mini Nutritional Assessment Short Form

Screening for malnutrition or risk of malnutrition will be carried out at 1 month (optional), 3, and 6 months after hospital discharge, using the Mini Nutritional Assessment Short Form (MNA-SF). The MNA-SF, consisting of 6 questions, is cost-effective, simple, and suitable for use in hospitals and therefore fulfills the requirements for a screening instrument, which can be used to identify existing malnutrition or the risk of malnutrition in older people [31]. The MNA-SF includes questions on mobility, psychological stress, or acute illness and neuropsychological problems, which increase the risk of malnutrition but do not directly detect malnutrition. The MNA-SF is therefore particularly suitable if the screening aims to detect malnutrition as early as possible [32].

#### Handgrip Strength

There is strong evidence for a correlation between reduced handgrip strength (HGS) and poor nutritional status, increased mortality, complication rates, and hospital length of stay [33-36]. Therefore, HGS will be measured 1 month (optional), 3, and 6 months after hospital discharge, using a dynamometer as follows: participants sitting in a chair with the elbow joint bent 90 degrees. On request, the dynamometer is squeezed as firmly as possible. First, the nondominant hand and then the dominant hand will be tested 3 times each. The highest value for each hand is entered into the database.

#### Refrigerator Door Sensors

**Figure 1:**
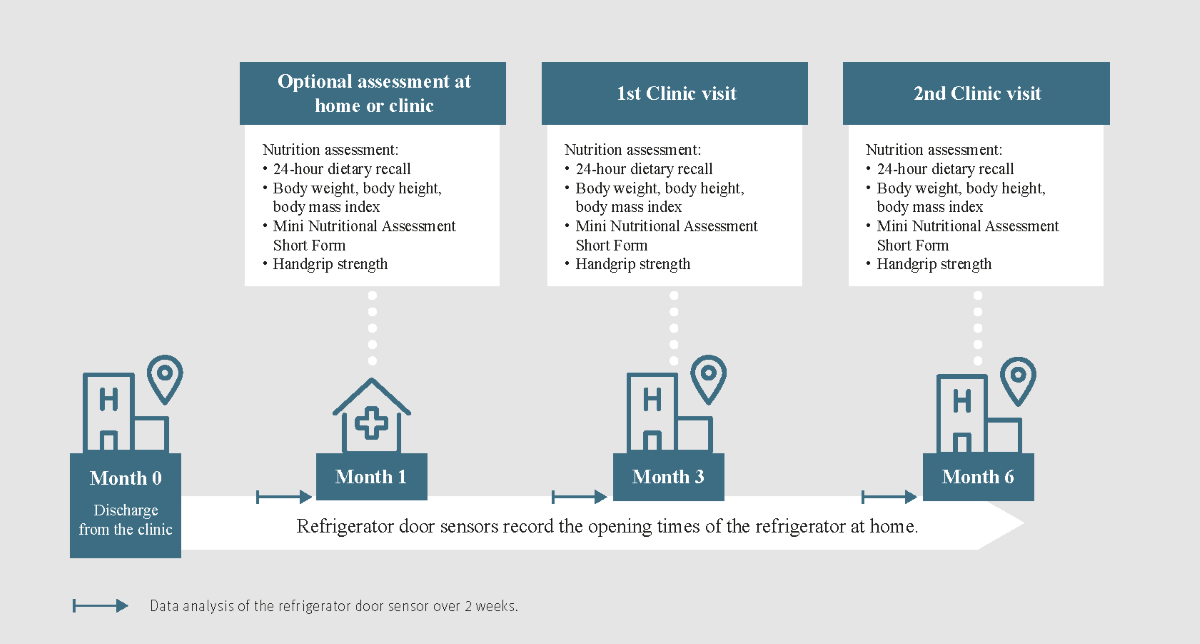
Assessments during the substudy.

The refrigerator at the patient’s home will be equipped with a door sensor recording the opening and closing events of the fridge door. This sensor consists of a small magnet fixed on the door and a sensor with a reed relay attached to the frame. When the door opens or closes, the sensor immediately sends this information to a base unit through an encrypted radio frequency protocol continuously with a resolution of 1 second. The base unit collects the data and sends it to the cloud in real time using the Global System of Mobile Communication networks. The duration of each refrigerator opening is calculated as the time difference between the opening and closing events. The system has been described in detail in the main study, where the sensor system is used to collect digital markers beyond refrigerator openings with the aim of evaluating the system’s potential to detect HF decompensation [27]. The integration of the magnetic refrigerator door sensor into the sensor system to detect refrigerator openings represents a minor extension to the overall setup. The high reliability of the ambient sensor system, with extremely low susceptibility of the sensor and the transmission system, has been shown previously [27]. The refrigerator door sensors will be installed at the patient’s home within 1 week after hospital discharge. Frequency and timing of refrigerator openings are collected over a 6-month period after hospital discharge. The assessment during the study is shown in Figure 1.

### Statistical Analysis

We will compute the cross-sectional correlations between protein intake (g) and first refrigerator door opening after midnight in minutes (RDmin) at the start (1 month) and at 3 and 6 months (primary outcome). Further, quantities of interest are the correlation between protein intake (g) and refrigerator opening frequency per day (RDfreq), between energy intake (kcal) and RDmin and RDfreq, between (risk of) malnutrition (score) and RDmin and RDfreq, and between HGS (kg) and RDmin and RDfreq. From the time series of refrigerator opening time after midnight in minutes over the period of 6 months, we will create 3 time windows, each window representing the 2-week period prior to each nutrition assessment (Figure 1). For each window, a mean RDmin measurement will be calculated, resulting in 3 RDmin measurements, 3 RDfreq, and 3 measurements of protein intake (g) and energy intake (kcal) from the 24-hour dietary recalls, 3 measurements of malnutrition and HGS (after 1, 3, and 6 months). We will estimate the cross-sectional associations between RDmin and RDfreq and protein intake (g), energy intake (kcal), (risk of) malnutrition (score), and HGS (kg) at 1, 3, and 6 months to quantify the correlation and to assess the stability of this correlation over time. We will compute Spearman correlation coefficients with corresponding 95% CIs.

### Ethical Considerations

The study will be conducted in accordance with the principles of the Declaration of Helsinki, the International Council for Harmonisation–Good Clinical Practice (ICH-GCP) Guideline, and the Human Research Act after protocol approval by the regional ethics committee (Ethikkommission Nordwestschweiz: BASEC-ID 2023-02138). Patients will provide written informed consent for the main and the substudy. Data will be collected by specially trained research staff and entered into a password-protected data environment. Each patient will be assigned a study-specific patient identification number (PID). For statistical analysis, datasets will be merged using the PID as an identifier. Upon completion of data collection, including follow-up, patient data will be coded using the PID, and the database will be locked. Coding using the PID will be done at the earliest time point after completion of follow-up data collection. Data generation, transmission, storage, and analysis of health-related personal data will comply with current Swiss legal requirements for data protection and will be performed according to the Human Research Ordinance Art. 5. Health-related personal data captured during this study will remain strictly confidential and will not be disclosed to third parties. Participants will be reimbursed for travel expenses for hospital follow-up visits. No costs will be incurred by participants for taking part in the study.

## Results

The study is in the collection phase. Study recruitment started in February 2024. Data analysis is scheduled to start after all data are collected. As of June 2025, 12 patients have been recruited. Results are expected to be published by the end of 2025.

## Discussion

### What Does the Study Offer?

Because overall diet quality is associated with the risk of frailty, and insufficient protein intake leads to loss of muscle mass and strength [13,14], more attention should be paid to healthy nutrition and adequate protein intake in patients with HF. Although results from other studies suggest that patients with HF may benefit from a high-protein diet, conclusive evidence from prospective controlled clinical trials, particularly in relation to mortality, is scarce. Individual studies, such as a randomized double-blind pilot study in 29 patients with HF, have shown improvements in quality of life after 18 weeks on a high-calorie, high-protein diet [37]. However, most studies on protein intake are observational and have been conducted in the general population [38], while limited data are available for patients with HF. Simpler and more innovative tools for collecting information on protein intake in patients with HF could reduce the burden of long-term home monitoring. Furthermore, such tools may help minimize recall bias, which is particularly problematic in older adults when using retrospective dietary assessment methods. Refrigerator openings could represent such a tool, both in terms of the first opening of the day and overall frequency. For example, a late first refrigerator opening after midnight may be indicative of skipping breakfast. In general, little is known about the nutritional status of older people who skip breakfast, and there are contradictory results regarding meal frequency and frailty [39,40].

To the best of our knowledge, this is the first prospective study investigating the use of refrigerator openings as a potential indicator of protein intake. The use of the refrigerator openings in relation to frailty and sarcopenia would allow easy monitoring. Interventions could be initiated at an early stage and thereby reduce disease progression.

### Limitations

This study has several limitations. First, the patterns of refrigerator openings—regarding time of day and frequency—may be subject to great variations, and the use of single values or combinations of values will require both internal and external validation. Second, the study is limited to patients who live alone. This complicates the generalizability of the results to other populations with HF. The small sample size is based on experience from a previous study, including a similar patient population with hospitalization for HF decompensation. At present, no prior data are available to support a formal power calculation. Third, to verify the accuracy of the refrigerator openings in assessing the protein intake, results must be compared with a standard reference method. This is done in this study with a 24-hour recall. As a retrospective method, it also has weaknesses, in particular, recall bias or unusual eating days. For this reason, at least two 24-hour dietary recalls per person are carried out.

### Conclusions

This study aims to provide new insight into the relation between refrigerator openings and nutritional intake, particularly protein intake. It is hypothesized that monitoring refrigerator openings within the framework of an ambient sensor system may help identify patients at risk for malnutrition and, as a result, for muscle mass and strength loss. Such identification of insufficient, and particularly protein-deficient, diets could enable simple monitoring and timely intervention to prevent or at least delay sarcopenia and frailty. This, in turn, may improve quality of life and potentially reduce health care costs. In addition, this low-effort monitoring approach could be of interest for other target groups.

## Appendix

Multimedia Appendix 1 Examples from the photo book of the national nutrition survey in Switzerland (menuCH).

## Abbreviations

AMPM: Automated Multiple-Pass Method
HF: heart failure
HGS: hand grip strength
ICH-GCP: International Council for Harmonisation–Good Clinical Practice
menuCH: National Nutrition Survey Switzerland
MNA: Mini Nutritional Assessment
MNA-SF: Mini Nutritional Assessment Short Form
PHQ-9: 9-item Patient Health Questionnaire
PID: patient identification number
RDfreq: refrigerator door opening frequencies per day
RDmin: refrigerator door opening after midnight in minutes

## Data Availability

The datasets generated and analyzed during this study are available from the corresponding author on reasonable request.

## References

[1] Bundesamt für Statistik (BFS) (2020). Szenarien zur Bevölkerungsentwicklung der Schweiz und der Kantone: 2020-2050.

[2] Gingrich A, Volkert D, Kiesswetter E, Thomanek M, Bach S, Sieber CC, Zopf Y (2019). Prevalence and overlap of sarcopenia, frailty, cachexia and malnutrition in older medical inpatients. BMC Geriatr.

[3] Ligthart-Melis GC, Luiking YC, Kakourou A, Cederholm T, Maier AB, de van der Schueren MAE (2020). Frailty, sarcopenia, and malnutrition frequently (co-)occur in hospitalized older adults: a systematic review and meta-analysis. J Am Med Dir Assoc.

[4] Jeejeebhoy KN (2012). Malnutrition, fatigue, frailty, vulnerability, sarcopenia and cachexia: overlap of clinical features. Curr Opin Clin Nutr Metab Care.

[5] Lardiés-Sánchez B, Sanz-París A, Dionyssiotis Y (2017). Sarcopenia and malnutrition in the elderly. Frailty and Sarcopenia - Onset, Development and Clinical Challenges.

[6] Hu X, Zhang L, Wang H, Hao Q, Dong B, Yang M (2017). Malnutrition-sarcopenia syndrome predicts mortality in hospitalized older patients. Sci Rep.

[7] Beaudart C, Sanchez-Rodriguez D, Locquet M, Reginster J, Lengelé L, Bruyère O (2019). Malnutrition as a strong predictor of the onset of sarcopenia. Nutrients.

[8] Sato PHR, Ferreira AA, Rosado EL (2020). The prevalence and risk factors for sarcopenia in older adults and long-living older adults. Arch Gerontol Geriatr.

[9] Verlaan S, Ligthart-Melis GC, Wijers SLJ, Cederholm T, Maier AB, de van der Schueren MAE (2017). High prevalence of physical frailty among community-dwelling malnourished older adults-a systematic review and meta-analysis. J Am Med Dir Assoc.

[10] Boulos C, Salameh P, Barberger-Gateau P (2016). Malnutrition and frailty in community dwelling older adults living in a rural setting. Clin Nutr.

[11] Zhang Y, Zhang J, Ni W, Yuan X, Zhang H, Li P, Xu J, Zhao Z (2021). Sarcopenia in heart failure: a systematic review and meta-analysis. ESC Heart Fail.

[12] Vitale C, Spoletini I, Rosano GM (2018). Frailty in heart failure: implications for management. Card Fail Rev.

[13] Kojima G, Avgerinou C, Iliffe S, Walters K (2018). Adherence to mediterranean diet reduces incident frailty risk: systematic review and meta-analysis. J Am Geriatr Soc.

[14] Cruz-Jentoft AJ, Dawson Hughes B, Scott D, Sanders KM, Rizzoli R (2020). Nutritional strategies for maintaining muscle mass and strength from middle age to later life: a narrative review. Maturitas.

[15] Coelho-Junior HJ, Marzetti E, Picca A, Cesari M, Uchida MC, Calvani R (2020). Protein intake and frailty: a matter of quantity, quality, and timing. Nutrients.

[16] Coelho-Junior HJ, Calvani R, Azzolino D, Picca A, Tosato M, Landi F, Cesari M, Marzetti E (2022). Protein intake and sarcopenia in older adults: a systematic review and meta-analysis. Int J Environ Res Public Health.

[17] Jyväkorpi SK, Pitkälä KH, Puranen TM, Björkman MP, Kautiainen H, Strandberg TE, Soini HH, Suominen MH (2016). High proportions of older people with normal nutritional status have poor protein intake and low diet quality. Arch Gerontol Geriatr.

[18] Mueller C (2015). Nutrition assessment and older adults. Top Clin Nutr.

[19] Schütz N, Knobel SEJ, Botros A, Single M, Pais B, Santschi V, Gatica-Perez D, Buluschek P, Urwyler P, Gerber SM, Müri RM, Mosimann UP, Saner H, Nef T (2022). A systems approach towards remote health-monitoring in older adults: introducing a zero-interaction digital exhaust. NPJ Digit Med.

[20] Struijk EA, Fung TT, Rodríguez-Artalejo F, Bischoff-Ferrari HA, Hu FB, Willett WC, Lopez-Garcia E (2022). Protein intake and risk of frailty among older women in the Nurses' Health Study. J Cachexia Sarcopenia Muscle.

[21] Zhang Z, Tan J, Luo Q (2024). Associations between breakfast skipping and outcomes in neuropsychiatric disorders, cognitive performance, and frailty: a Mendelian randomization study. BMC Psychiatry.

[22] Kinoshita K, Satake S, Murotani K, Li J, Yasuoka M, Arai H (2023). Breakfast skipping and frailty: a cross-sectional study in community-dwellers aged 75 years or over. Geriatr Gerontol Int.

[23] (2024). Bestand der Haushalte und experimentelle Statistik STATPOP im Jahr 2023. Bundesamt für Statistik.

[24] Gassmann K-G, Rupprecht R, Freiberger E, Study Group IZG (2009). Predictors for occasional and recurrent falls in community-dwelling older people. Z Gerontol Geriatr.

[25] Ek S, Rizzuto D, Calderón-Larrañaga A, Franzén E, Xu W, Welmer A (2019). Predicting first-time injurious falls in older men and women living in the community: development of the first injurious fall screening tool. J Am Med Dir Assoc.

[26] Braendle K, Egli A, Bischoff-Ferrari H, Freystaetter G (2024). Does living alone influence fall risk among Swiss older adults aged 60+? A pooled observational analysis of three RCTs on fall prevention. BMJ Open.

[27] Vögeli B, Arenja N, Schütz N, Nef T, Buluschek P, Saner H (2024). Evaluation of ambient sensor systems for the early detection of heart failure decompensation in older patients living at home alone: protocol for a prospective cohort study. JMIR Res Protoc.

[28] Willett W (2013). Nutritional Epidemiology.

[29] Moshfegh AJ, Rhodes DG, Baer DJ, Murayi T, Clemens JC, Rumpler WV, Paul DR, Sebastian RS, Kuczynski KJ, Ingwersen LA, Staples RC, Cleveland LE (2008). The US Department of Agriculture automated multiple-pass method reduces bias in the collection of energy intakes. Am J Clin Nutr.

[30] menuCH methodik. Bundesamt für Lebensmittelsicherheit und Veterinärwesen (BLV).

[31] Kaiser MJ, Bauer JM, Ramsch C, Uter W, Guigoz Y, Cederholm T, Thomas DR, Anthony P, Charlton KE, Maggio M, Tsai AC, Grathwohl D, Vellas B, Sieber CC, MNA-International Group (2009). Validation of the Mini Nutritional Assessment Short-Form (MNA-SF): a practical tool for identification of nutritional status. J Nutr Health Aging.

[32] Bundesamt für Lebensmittelsicherheit und Veterinärwesen (BLV) (2018). Eidgenössische Ernährungskommission. Ernährung im Alter - ein Expertenbericht der EEK.

[33] Norman K, Stobäus N, Gonzalez MC, Schulzke J, Pirlich M (2011). Hand grip strength: outcome predictor and marker of nutritional status. Clin Nutr.

[34] McNicholl T, Dubin JA, Curtis L, Mourtzakis M, Nasser R, Laporte M, Keller H (2019). Handgrip strength, but not 5-meter walk, adds value to a clinical nutrition assessment. Nutr Clin Pract.

[35] Guerra RS, Fonseca I, Pichel F, Restivo MT, Amaral TF (2015). Handgrip strength and associated factors in hospitalized patients. JPEN J Parenter Enteral Nutr.

[36] Bohannon RW (2008). Hand-grip dynamometry predicts future outcomes in aging adults. J Geriatr Phys Ther.

[37] Rozentryt P, von Haehling S, Lainscak M, Nowak JU, Kalantar-Zadeh K, Polonski L, Anker SD (2010). The effects of a high-caloric protein-rich oral nutritional supplement in patients with chronic heart failure and cachexia on quality of life, body composition, and inflammation markers: a randomized, double-blind pilot study. J Cachexia Sarcopenia Muscle.

[38] Song M, Fung TT, Hu FB, Willett WC, Longo VD, Chan AT, Giovannucci EL (2016). Association of animal and plant protein intake with all-cause and cause-specific mortality. JAMA Intern Med.

[39] Yokoyama Y, Kitamura A, Nishi M, Seino S, Taniguchi Y, Amano H, Ikeuchi T, Shinkai S (2019). Frequency of balanced-meal consumption and frailty in community-dwelling older Japanese: a cross-sectional study. J Epidemiol.

[40] Johannesson J, Rothenberg E, Gustafsson S, Slinde F (2019). Meal frequency and vegetable intake does not predict the development of frailty in older adults. Nutr Health.

