# Supplementary material for: Association Between Refrigerator Openings and Protein Intake After Hospitalization for Heart Failure Decompensation: Protocol for a Prospective Cohort Pilot Study
Source: JMIR Res Protoc. 2025 Aug 18;14:e66299. doi: 10.2196/66299 (PMC12402733; doi:10.2196/66299)
Supplement: Multimedia Appendix 1 [file resprot_v14i1e66299_app1.pdf]

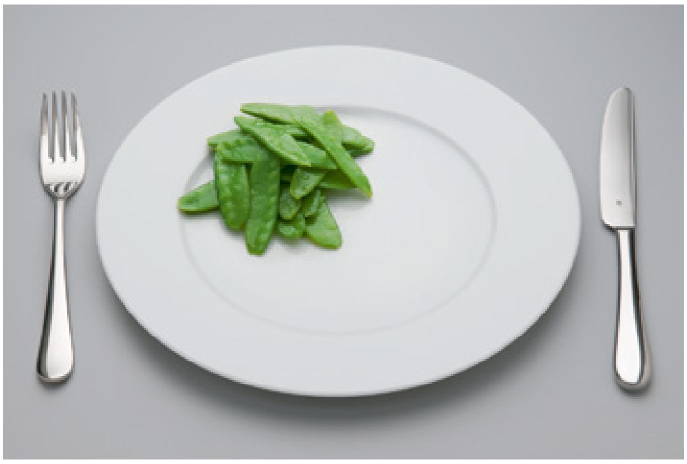

808 - 1

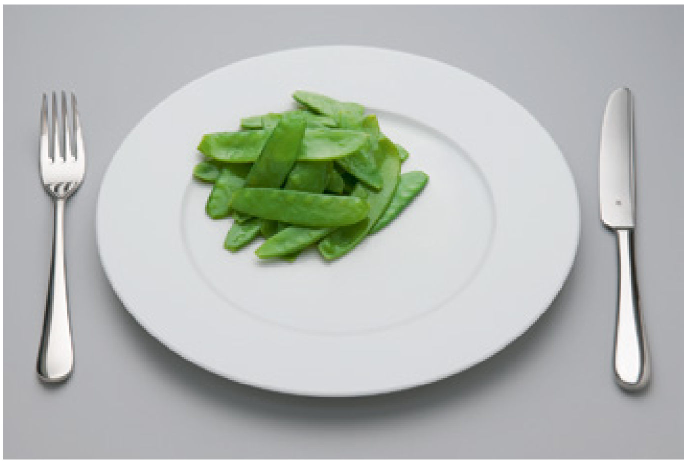

808 - 2

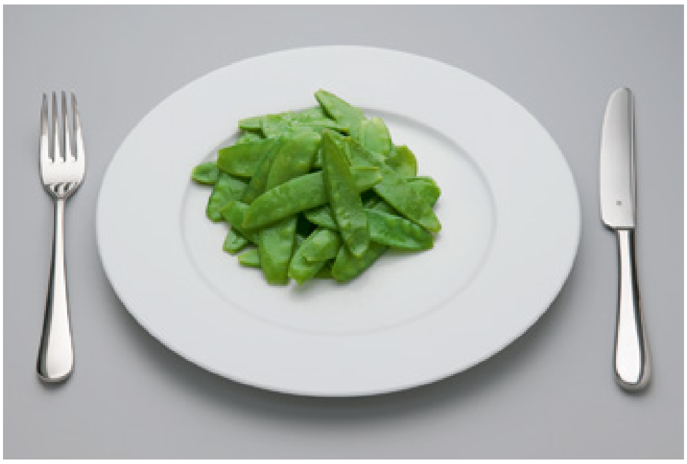

808 - 3

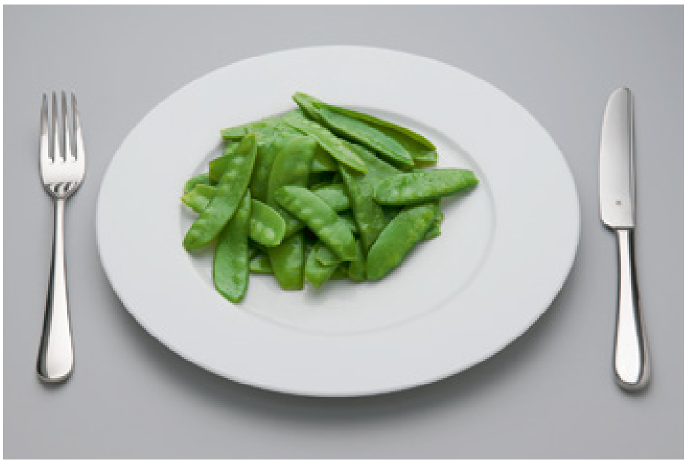

808 - 4

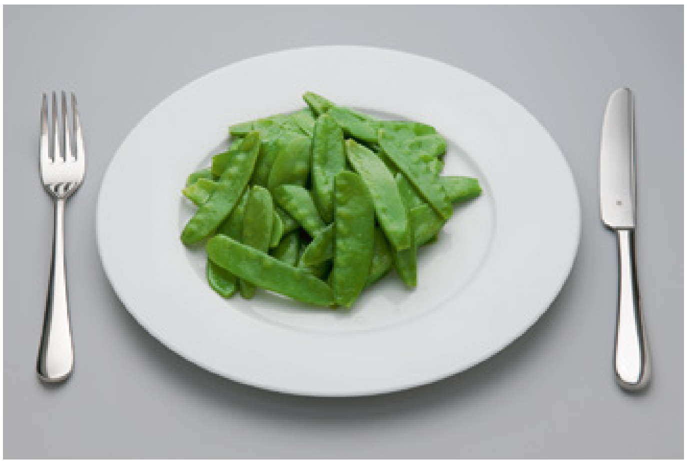

808 - 5

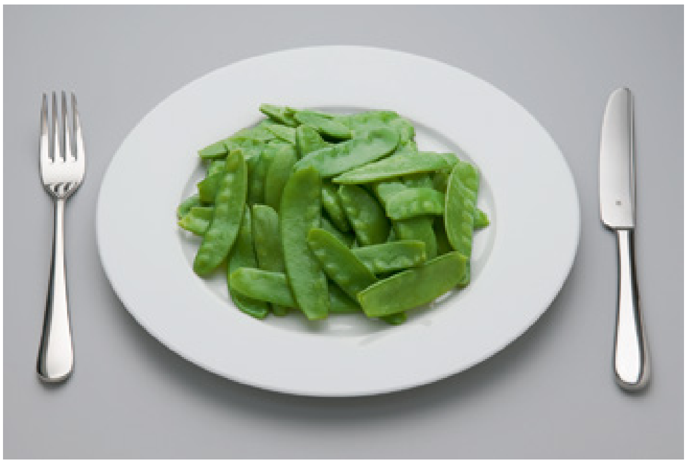

808 - 6

Lasagne

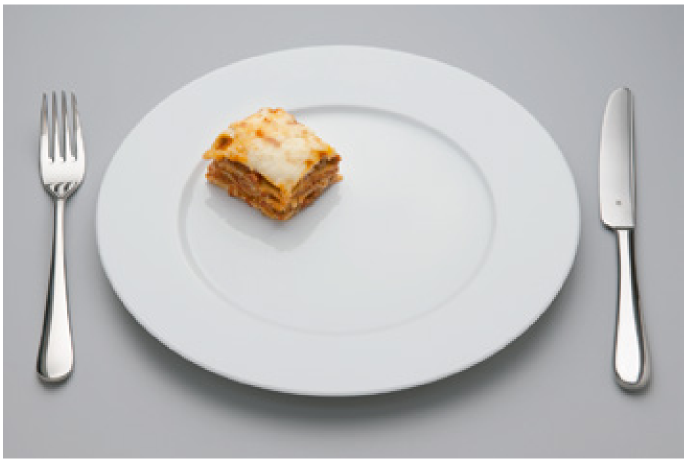

820 - 1

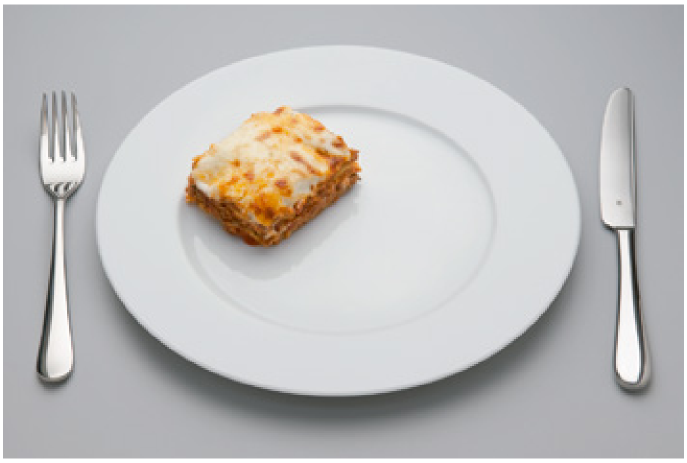

820 - 2

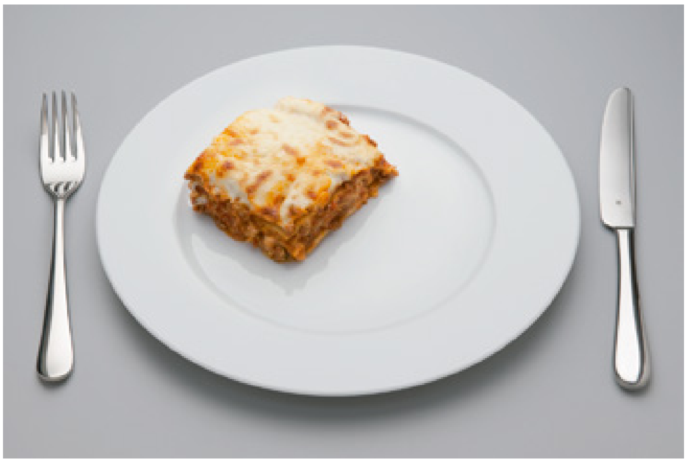

820 - 3

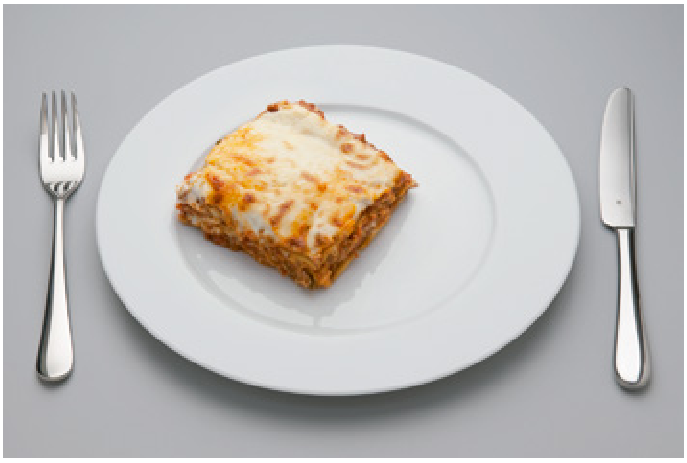

820 - 4

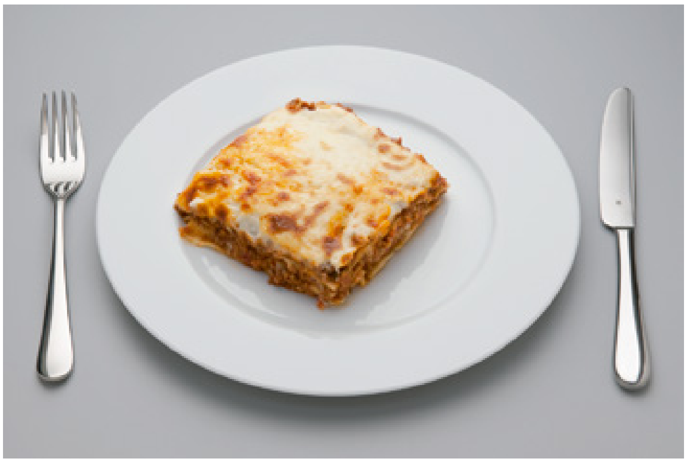

820 - 5

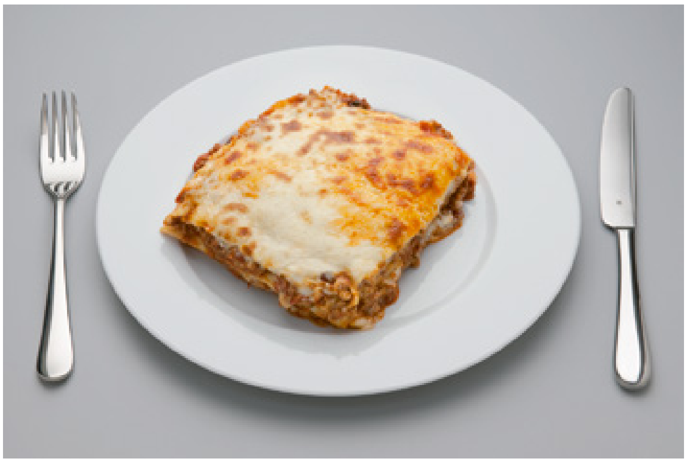

820 - 6

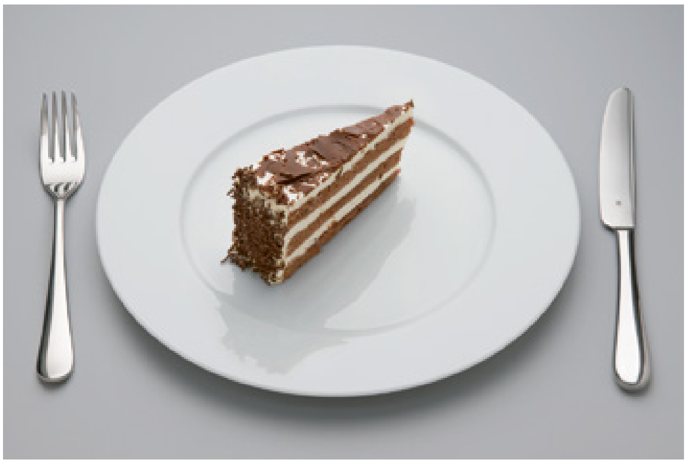

846 - 1

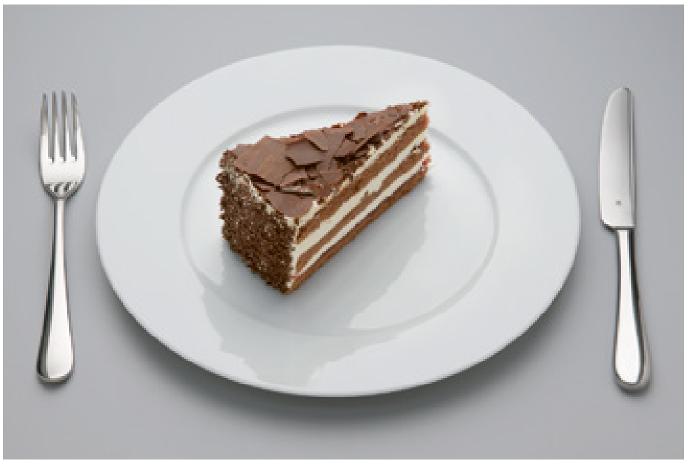

846 - 2

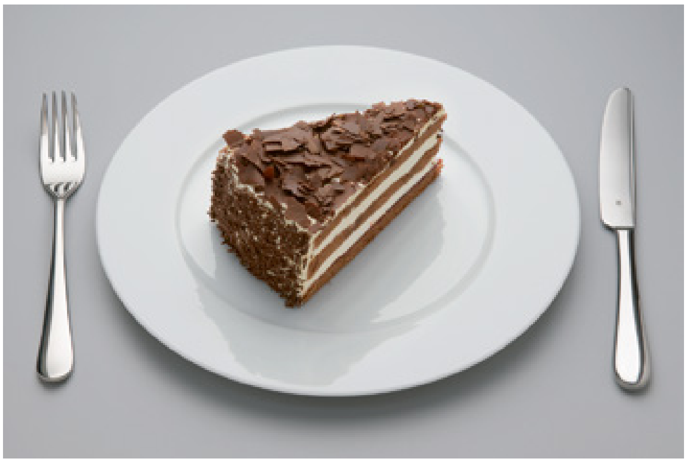

846 - 3

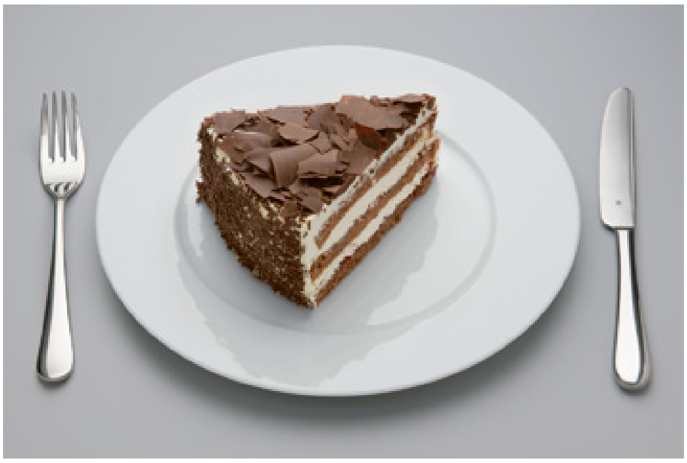

846 - 4

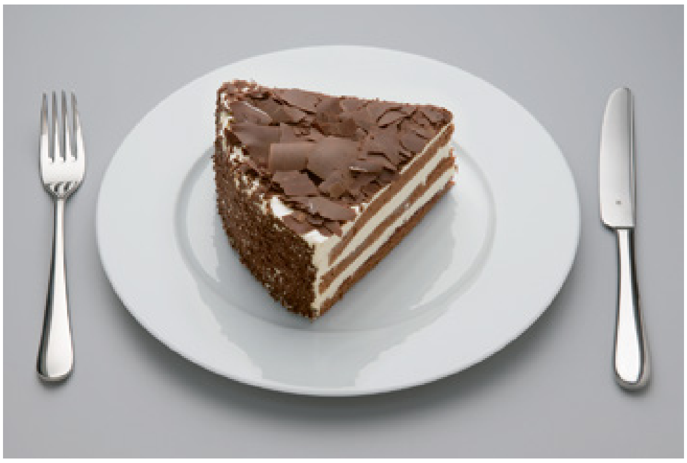

846 - 5

Impressum

Herausgeber / Éditeur / Editore
Eidgenössisches Departement des Innern (EDI)
Bundesamt für Lebensmittelsicherheit und Veterinärwesen (BLV)

Département fédéral de l'intérieur (DFI)
Office fédéral de la sécurité alimentaire et des affaires vétérinaires (OSAV)

Dipartimento federale dell'interno (DFI)
Ufficio federale della sicurezza alimentare e di veterinaria (USAV)

Kontakt / Contact / Contatto
Bundesamt für Lebensmittelsicherheit und Veterinärwesen (BLV)
Postfach
CH-3003 Bern
info@blv.admin.ch
www.blv.admin.ch

Bildnachweis / Crédit photographique / Crediti fotografici
1-517: 'EPIC-Soft picture book for the estimation of food portion sizes'
(Van Kappel AL, Amoyel J, Slimani N, Vozar B, Riboli E. © IARC, 1995)

Gestaltung / Mise en page / Progettazione
BOLD AG, Bern

Bild-Layout / Mise en page des images / Layout di immagine
Websurface, Obfelden

Fotos / Photos / Foto
Patrick Rohner, Bennau

Zitierweise / Mode de citation / Modalità di citazione / Citation
Federal Food Safety and Veterinary Office (FSVO). Picture book menuCH-Kids: Swiss manual to estimate food portion sizes. Bern: FSVO; 2023.

Gedruckt auf recycling papier / Imprimé sur du papier recyclé / Stampato su carta riciclata

© Bundesamt für Lebensmittelsicherheit und Veterinärwesen (BLV)
